# Adiporedoxin suppresses endothelial activation via inhibiting MAPK and NF-κB signaling

**DOI:** 10.1038/srep38975

**Published:** 2016-12-12

**Authors:** Hui He, Fang Guo, Yong Li, Fatma Saaoud, Brooks D. Kimmis, Jeena Sandhu, Michelle Fan, Dev Maulik, Susan Lessner, Christopher J. Papasian, Daping Fan, Zhisheng Jiang, Mingui Fu

**Affiliations:** 1 Institute of Cardiovascular Disease, Key Laboratory for Arteriosclerology of Hunan Province, University of South China, Hengyang, Hunan 421001, China; 2 Department of Basic Medical Science, Shock/Trauma Research Center, School of Medicine, University of Missouri Kansas City, Kansas City, MO 64108, USA; 3 Institute of Translational Medicine, Nanchang University, Nanchang, Jiangxi, 330031, P. R. China; 4 Department of Cell Biology and Anatomy, University of South Carolina School of Medicine, Columbia, SC 29209, USA; 5 Department of Obstetrics and Gynecology, University of Missouri - Kansas City School of Medicine, Kansas City, MO 64108, USA

## Abstract

Adiporedoxin (Adrx) is a recently discovered redox regulatory protein that is preferentially expressed in adipose tissue and plays a critical role in the regulation of metabolism via its modulation of adipocyte protein secretion. We here report that Adrx suppresses endothelial cell activation via inhibiting MAPK and NF-kB signaling pathways. Adrx is constitutively expressed in human vascular endothelial cells, and significantly induced by a variety of stimuli such as TNFα, IL-1β, H_2_O_2_ and OxLDL. Overexpression of Adrx significantly attenuated TNFα-induced expression of VCAM-1 and ICAM-1, and thus reduced monocyte adherence to human umbilical vein endothelial cells (HUVECs). Conversely, siRNA-mediated knockdown of Adrx increased TNFα-induced expression of adhesion molecules and monocyte adherence to HUVECs. Furthermore, forced expression of Adrx decreased TNFα-induced activation of ERK1/2, JNK, p38 and IKKs in HUVECs. Adrx mutant in the CXXC motif that lost its anti-redox activity is less efficient than the wild-type Adrx, suggesting that Adrx-mediated inhibition of endothelial activation is partially dependent on its antioxidant activity. Finally, Adrx expression was markedly increased in human atheroma compared with normal tissue from the same carotid arteries. These results suggest that Adrx is an endogenous inhibitor of endothelial activation, and might be a therapeutic target for vascular inflammatory diseases.

Endothelial cell activation plays a key role in the pathogenesis of atherosclerosis and other vascular diseases^1^. Accordingly, regulating inflammatory activation of vascular endothelial cells is a potential therapeutic strategy for treating chronic inflammatory diseases, such as atherosclerosis. A crucial step in chronic inflammation is the recruitment and transendothelial migration of monocytes from the circulation into the subendothelial space of large arteries, where they differentiate into macrophages and become functionally active^2^. These processes are precisely controlled by cytokines such as interleukin-1β (IL-1β), IL-6, IL-8 and tumor necrosis factor α (TNFα), which stimulate endothelial cell expression of adhesion molecules and chemokines^3^,^4^. These latter molecules attract leukocytes to the vascular wall, promoting inflammation and atherogenesis^3^. TNFα activation of these pathways requires generation of reactive oxygen species (ROS) that promote kinase activation and phosphatase inactivation^5^. MAPK and NF-κB, which are involved in these pathways, have been well-studied, but regulation of these pathways is not completely understood.

Adiporedoxin (Adrx), also known as PAMM (peroxiredoxin-like 2 activated in M-CSF stimulated monocytes), is a 24-kD redox regulatory protein containing a CXXC-type PRX-like 2 domain that is critical for its redox regulatory activity^6^. It was previously reported that Adrx modulates osteoclast differentiation, plays a critical role in adipocyte biology, and regulates metabolism, at least in part, by modulating protein secretion (e.g. adiponectin) by adipocytes^6^,^7^. Adrx deficient mice have reduced levels of circulating adiponectin, and have been found to be moderately hyperinsulinemic. Moreover, adipose tissue from Adrx knockout mice is virtually free of fibrosis, and these mice exhibit a complex phenotypes tending towards insulin resistance^7^. Importantly, our previous work showed that Adrx protein, which is secreted by adipocytes, has anti-inflammatory effects on activated macrophages via both intracellular and extracellular mechanisms^8^.

In the present study, we sought to investigate the role of Adrx in endothelial cell activation. The data presented here suggest that Adrx is a negative regulator of cytokine-induced MAPK and NF-κB signaling pathways in human vascular endothelial cells, by which it controls TNFα-induced expression of adhesion molecules and monocyte adherence to endothelial cells. Further, the expression of Adrx is markedly increased in human atheroma, suggesting that Adrx may serve to suppress atherogenesis in humans.

## Results

### Adrx is induced by a variety of stimuli in human vascular endothelial cells

**Figure 1:**
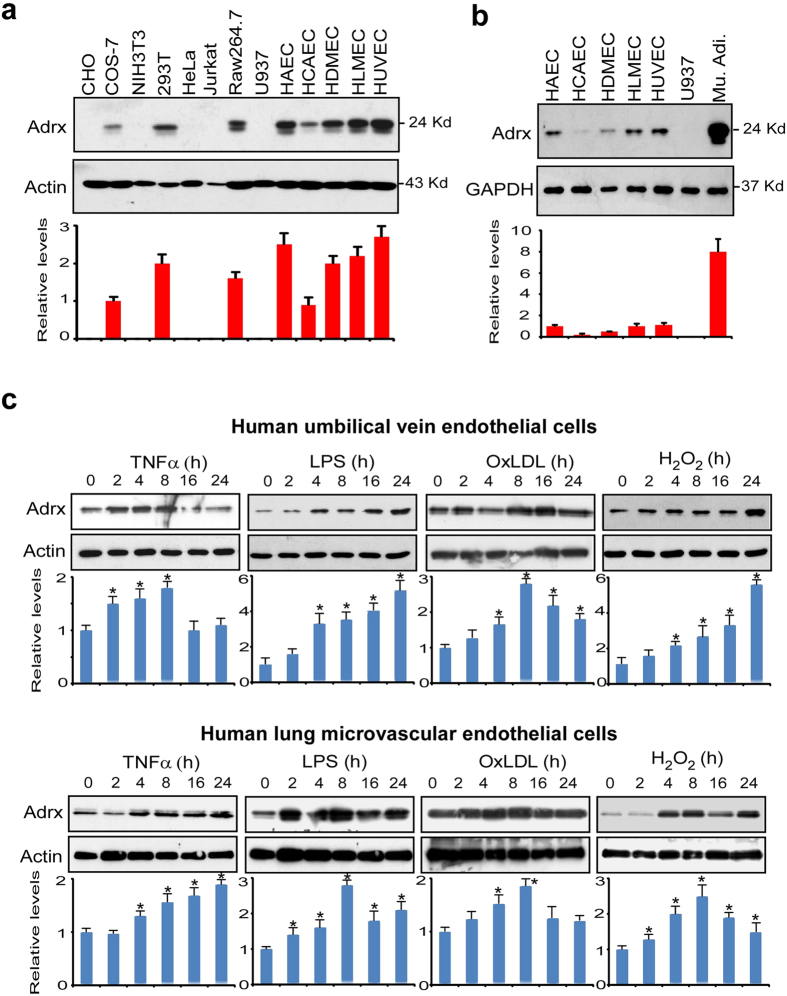
Expression of Adrx in human vascular endothelial cells. (**a,b)** Cell lysates were harvested from human primary vascular endothelial cells including HAEC, HCAEC, HDMEC, HLMEC, HUVEC and other cell lines as indicated. Mouse adipocytes (Mu.Adi) were generated from 3T3L-1 subjected to a differentiation program for 10 days. Western blot was performed to detect Adrx protein levels in these cell lines. Actin or GAPDH was probed as loading control. (**c**) HUVEC and (**d**) HLMEC were stimulated with various stimuli including TNFα, LPS, OxLDL and H_2_O_2_ for different durations as indicated. Cell lysates were harvested for Western blot analysis. The bands of Western blot were quantified by Gel-Pro Analyzer software and presented as fold changes at the bottom of the images. The quantified data from three independent experiments and analyzed by two-way ANOVA followed by Bonferroni post-test. *p < 0.05 vs 0 time point.

We previously showed that Adrx suppressed the response of macrophages to proinflammatory stimuli^8^. To investigate the role of Adrx in vascular endothelial cells, we first examined the expression of Adrx in a variety of non- endotheial cell lines and compared them to endothelial cell lines, including human aortic endothelial cells (HAEC), human coronary artery endothelial cells (HCAEC), human dermal microvascular endothelial cells (HDMEC), human lung microvascular endothelial cells (HLMEC) and HUVEC. We found that Adrx protein was enriched in all human endothelial cells except HCAECs. It was moderately expressed in HCAECs as well as in COS-7, 293 T and Raw267.4 cells, but was not expressed in CHO, NIH3T3, HeLa, Jurkat or U937 cell lines (Fig. 1a). To compare Adrx expression in human endothelial cells with that in adipocytes, we differentiated 3T3-L1 into mature adipocytes and harvested cell extracts (human adipocytes were not available). As shown in Fig. 1b, Adrx protein was abundant in adipocytes and is also constitutively expressed (about 15% compared to adipocytes) in human endothelial cells. In addition, we found that Adrx expression was strongly upregulated by a variety of stimuli such as TNFα, LPS, oxLDL and H_2_O_2_ in both HUVECs and HLMECs (Fig. 1c) in a time-dependent manner, though their expression patterns were not exactly the same. In HUVECs, TNFα and OxLDL induced Adrx expression peaked at 8 hours and then gradually declined to baseline at 24 hours. However, LPS and H_2_O_2_ increased Adrx expression and reached the peak at 24 hours. In HLMECs, LPS, OxLDL and H_2_O_2_ induced Adrx expression peaked at 8 hours and then gradually declined. TNFα increased Adrx expression and reached the peak at 24 hours. Taken together, our observations demonstrate that Adrx expression by vascular endothelial cells is enhanced by a variety of proinflammatory stimuli. Consequently, we hypothesized that Adrx regulates vascular endothelial activation in response to these stimuli

### Overexpression of Adrx inhibits the expression of adhesion molecules and monocyte adherence to HUVECs

**Figure 2:**
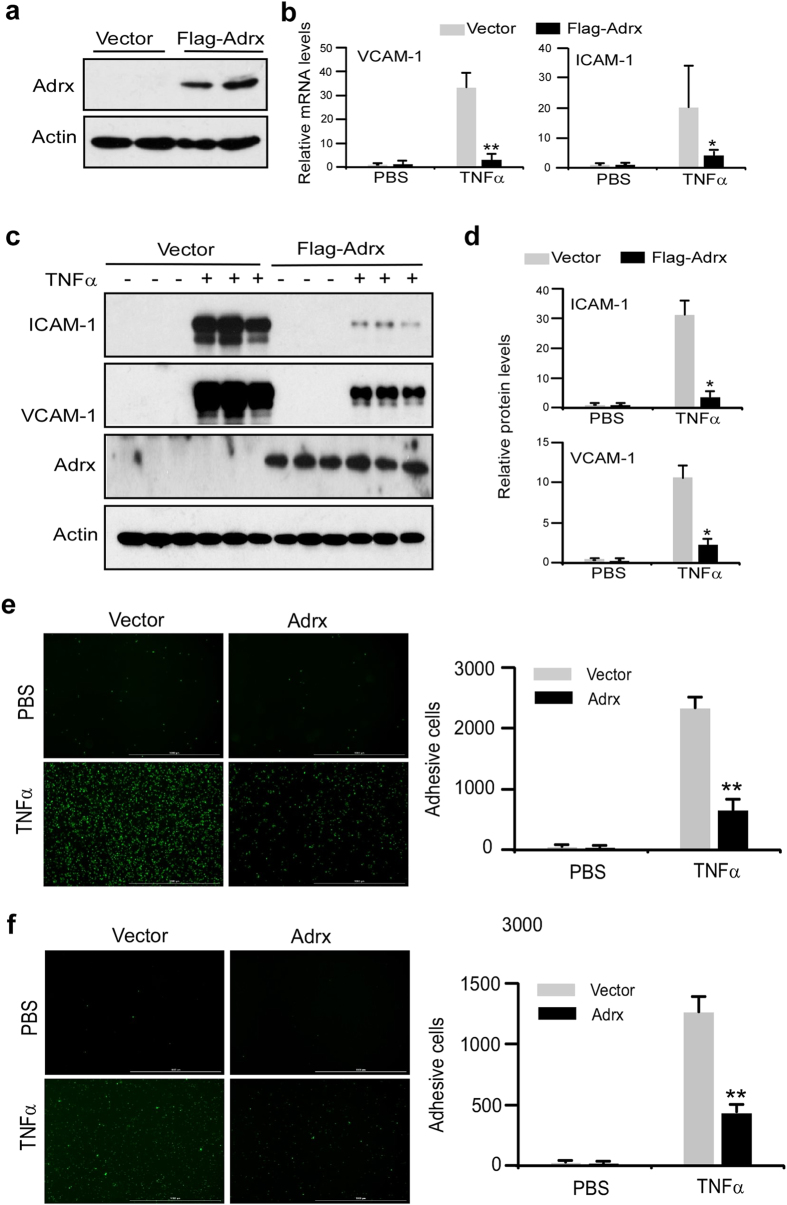
Overexpression of Adrx inhibited VCAM-1 and ICAM-1 gene expression and monocyte adherence to HUVEC. (**a**) HUVECs were transiently transfected with Flag-Adrx or empty vector for 24 hours. The cell lysates were harvested, and Adrx protein levels in the transfected cells were measured by Western blot with anti-Flag antibody. Actin was probed as loading control. (**b**) The transfected cells were treated with or without 10 ng/ml of TNF-α for 4 hours. The relative mRNA levels of VCAM-1 and ICAM-1 were detected by QPCR. Data presented represent mean ± SD; n = 4, *P < 0.05, **P < 0.01 vs vector group. (**c,d**) HUVEC were treated with or without TNFα for 8 hours and the protein levels of Adrx, VCAM-1, and ICAM-1were detected by Western blot. The bands of Western blot were quantified by Gel-Pro Analyzer software and presented as fold changes in (**d**); n = 3, *P < 0.05 vs vector group. (**e,f**) HUVECs were transiently transfected with Flag-Adrx or empty vector for 24 hours. Transfected cells were treated with or without 10 ng/ml of TNF-α for 8 hours and then cultured with PKH67-labeled THP-1 cells (**e**) or human primary monocytes (**f**). Thirty minutes later, floating cells were washed away and adhesive cells were imaged using a Cytation 3 Cell Imaging Multi-mode Reader (Biotek Instruments). Adhesive cells was counted and analyzed. Data represent three independent experiments. *P < 0.05, **P < 0.01.

To test our hypothesis that Adrx regulates vascular endothelial activation in response to proinfloammatory stimuli, we examined the effects of Adrx overexpression on TNFα-induced expression of adhesive molecules in HUVECs. First, we confirmed Adrx overexpression in transfected HUVECs by Western blot (Fig. 2a). Next, HUVECs were stimulated with, or without, TNFα for 8 hours after transfection with Flag-Adrx or empty vector, and cellular RNA and lysates were harvested from treated cells for Q-PCR and Western blot analysis. As shown in Fig. 2b, analysis by Q-PCR revealed that TNFα enhanced the expression of VCAM-1 and ICAM-1mRNA by more than twenty fold in HUVECs, and that overexpression of Adrx markedly attenuated TNFα–induced expression of VCAM-1 and ICAM-1 mRNA. Western blot analysis further confirmed that Adrx overexpression suppressed TNFα-induced VCAM-1 and ICAM-1 protein expression (Fig. 2c,d). To determine the functional consequence of Adrx’s effect on the expression of adhesive molecules, we examined the effect of Adrx on THP-1 monocyte adhesion to activated HUVECs. When HUVECs were stimulated with TNFα (10 ng/mL), THP-1 cell adhesion was substantially increased, and Adrx overexpression suppressed TNFα-induced adhesion of THP-1 cells to HUVECs by 82% (Fig. 2e). Similarly, Adrx overexpression also suppressed TNFα-induced adhesion of human primary monocytes to HUVECs (Fig. 2f). These colllective results support the concept that Adrx functions as a negative regulator of cytokine-induced inflammatory responses in vascular ECs.

### Knocking down of Adrx increased TNFα-induced endothelial cell activation

**Figure 3:**
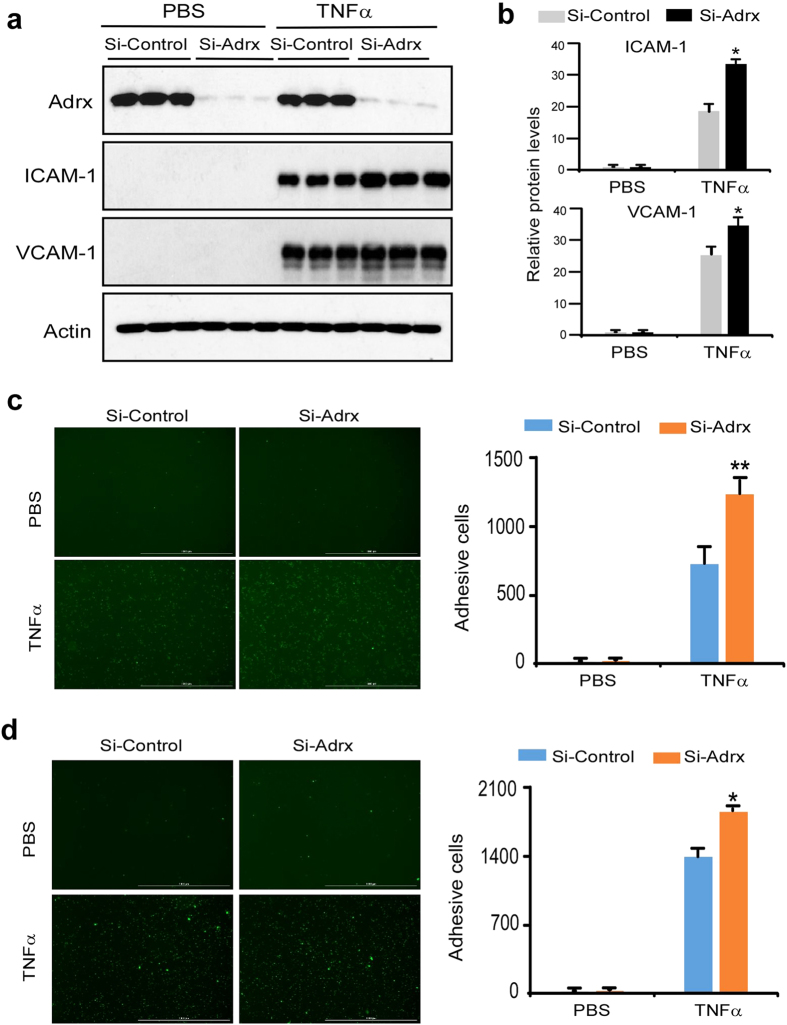
Adrx Knockdown augmented TNFα-induced expression of ICAM-1 and VCAM-1, and monocyte adherence to HUVEC. (**a)** HUVECs were transiently transfected with short interfering RNA targeting on Adrx (si-Adrx) or non-specific short interfering RNA (si-Control) by electroporation (Amaxa). Transfected cells were quiescent for 24 hours and then treated with or without TNFα for 8 hours. Expression of Adrx, ICAM-1 and VCAM-1 were detected using Western blot analysis. (**b)** Band intensity was quantified by Gel-Pro Analyzer software and normalized protein levels of ICAM-1 and VCAM-1 are shown in Fig. 4b; n = 3, *P < 0.05 vs treated si-Control group. (**c,d)** Transfected HUVECs were treated with or without 10 ng/ml TNFα for 8 hours and incubated with PHK67-labeled THP-1 cells (**c**) or human primary monocytes (**d**) for another 1 hour. Following washing, attached cells were visualized by Cytation 3 Cell Imaging Multi-mode Reader (Biotek Instruments). Adhesive cells were counted and analyzed. Data presented represent mean ± SD; n = 3. *P < 0.05 vs treated si-Control group.

To further study the role of Adrx in endothelial activation, we performed a loss-of-function study using the RNA interference technique. Western blot showed that transfection of HUVEC’s with Adrx siRNA substantially inhibited Adrx expression by 90% compared with cells transfected with control siRNA (Fig. 3a). As expected, TNFα treatment significantly induced expression of ICAM-1 and VCAM-1 in HUVECs treated with control siRNA. Compared to these controls, TNFα-induced expression of ICAM-1 and VCAM-1 was enhanced in HUVEC’s whose Adrx expression was knocked down by siRNA-treatment (Fig. 3a). Quantitative analysis of these bands showed that knock down of Adrx expression with siRNA significantly enhanced expression of ICAM-1 and VCAM-1 in HUVEC’s (Fig. 3b). The functional impact of enhancing ICAM-1 and VCAM-1 expression in HUVEC’s by knocking down Adrx expression with siRNA was demonstrated by examining THP-1 cell adhesion. siRNA-mediated knock-down of Adrx expression, consistently increased adhesion of THP-1 cells to TNFα-stimulated HUVECs by 70% (Fig. 3c). The adhesion experiments using human primary monocytes showed similar results to THPs (Fig. 3d). These results further support the concept that Adrx functions as a negative regulator of cytokine-induced inflammatory responses in vascular ECs.

### Overexpression of Adrx inhibited MAPK and NF-κB signal pathways

**Figure 4:**
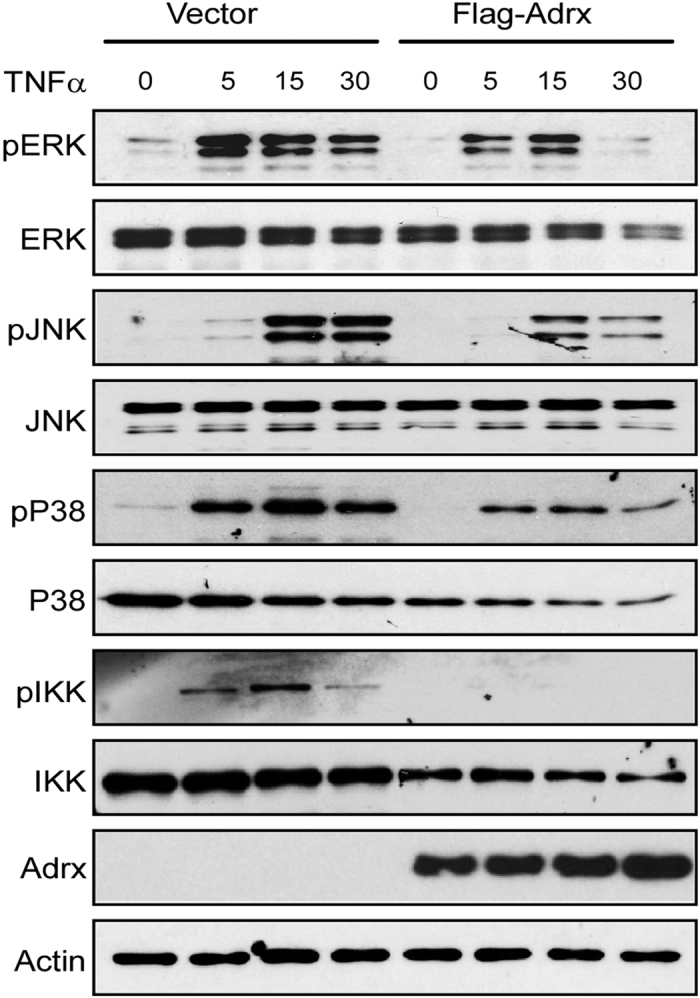
Overexpression of Adrx inhibited TNFα-induced MAPK and NF-κB signal pathways in HUVEC. HUVECs were transiently transfected with Flag-Adrx expression plasmid or empty vector. After 24 hours, transfected cells were treated with 10 ng/ml of TNFα for 0, 5, 15 and 30 minutes as indicated. Cell lysates were extracted and Western blots were performed to detect phosphorylation of ERK1/2, JNK, p38 and IKK with specific antibodies as indicated. Total protein levels of ERK1/2, p38, JNK and IKK were also detected. Experiments were repeated twice and showed consistent results.

The expression of endothelial adhesion molecules is mainly attributed to MAPK and NF-κB signaling pathways. To define the mechanisms by which Adrx suppresses expression of adhesive molecules in HUVECs, we analyzed the effect of Adrx overexpression on the activation of MAPK and NF-κB signal pathways. Briefly, HUVECs were first transfected with Flag-Adrx or empty vector. After 24 hours, transfected cells were stimulated with TNFα for different time intervals as indicated (Fig. 4). As expected, TNFα treatment induced the phosphorylation of ERK1/2, JNK, p38 and IKKα/β during these experimental intervals. Overexpression of Adrx significantly attenuated TNFα-induced phosphorylation of ERK1/2, JNK, p38 and IKKα/β in HUVECs. These results suggest that the mechanism by which Adrx negatively regulates endothelial cell activation in response to inflammatory stimuli, involves suppression of MAPK and NF-κB signaling pathways.

### Adrx-mediated inhibition on endothelial activation may depend on its anti-redox activity

**Figure 5:**
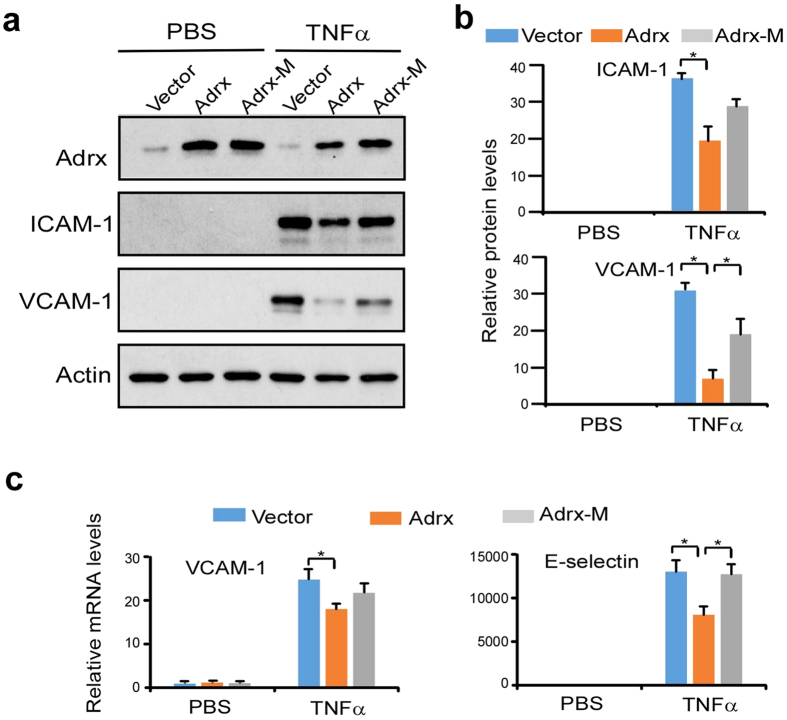
Adrx-mediated inhibition of endothelial activation may be dependent on its antioxidant activity. (**a)** HUVECs were transiently transfected with Adrx or Adrx mutant (C88G) expression plasmids, or empty vector. After 24 hours, transfected cells were treated with or without TNFα for 8 hours. Expression of Adrx, ICAM-1 and VCAM-1 were detected using Western blot analysis. (**b)** Band intensity was quantified by Gel-Pro Analyzer software and normalized protein levels of ICAM-1 and VCAM-1 are shown in (**b**) Experiments were repeated twice and showed consistent results. (**c**) Transfected cells were stimulated with or without TNFα for 4 hours. Relative mRNA levels of VCAM-1 and E-selectin were detected by QPCR. Actin levels were used to normalize. Data represent mean ± SD, n = 3, *P < 0.05.

As previous reported, Adrx is a redox regulatory protein whose anti-redox activity is dependent on its CXXC motif; mutation of the CXXC motif resulted in a loss of anti-redox activity^6^. To determine whether Adrx-mediated inhibition of endothelial activation is dependent on its anti-redox activity, we transfected expression plasmids for wild-type Adrx, mutant Adrx (Adrx-M), or empty vector, into HUVECs. Transfected cells were treated with TNFα for 8 hours. As expected, and shown in Fig. 5a, TNFα significantly induced the expression of both ICAM-1 and VCAM-1, and overexpression of Adrx reduced TNFα-induced expression of these adhesion molecules. However, overexpression of Adrx mutant compromised this effect, at least partially, compared to wild-type Adrx. Quantitative analysis showed TNFα induced expression of ICAM-1 and VCAM-1 was significantly higher in cells transfected with Adrx-M vs Adrx (Fig. 5b). Furthermore, QPCR analysis showed that Adrx expression decreased mRNA levels for both VCAM-1 and E-selectin, whereas Adrx-M failed to do so (Fig. 5c). These collective results suggest that Adrx-mediated inhibition of endothelial activation in response to proinflammatory stimuli may depend on its anti-redox activity.

### Adrx mRNA expression is significantly increased in atheromatous tissue from human arteries

**Figure 6:**
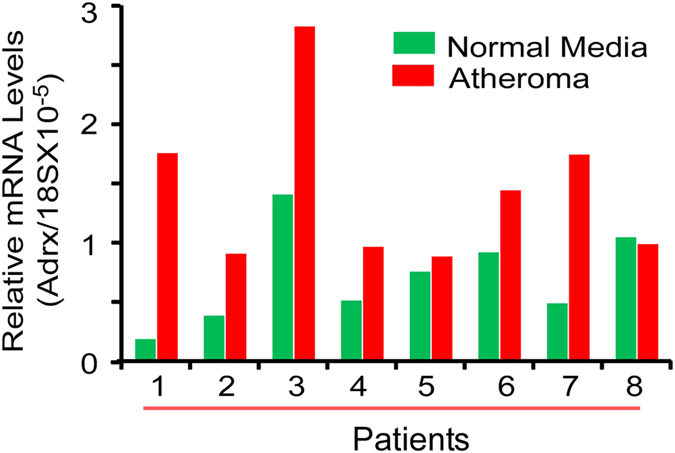
Expression analysis of Adrx in human atherosclerotic lesions. Normal and atheromatous tissues from carotid arteries from 8 patients who underwent carotid endarterectomy were used to measure Adrx expression levels by QPCR. Data were normalized by 18 S mRNA levels and presented as relative levels to 18 S. The average levels of Adrx in atheromatous tissues from eight patients was 2.8 fold compared that in normal carotid artery tissues and P = 0.02.

For this analysis, we collected tissue from 8 patients who underwent carotid endarterectomy. For all 8 patients, atheromatous tissue and normal tissue was collected. For each of these patients, total RNA was isolated from both tissues, and Adrx mRNA levels in normal vs. atheromatous tissue were compared by QPCR. As shown in Fig. 6, in most samples, Adrx mRNA levels were higher in atheromatous vs. normal tissue from 8 patients. The average levels of Adrx mRNA in atheroma was 2.8 folds compared to the levels in normal media.

## Discussion

The key finding from the current study is that Adrx functions as a negative regulator of cytokine-induced inflammatory responses in vascular EC. First, we demonstrated that Adrx was expressed in a variety of human endothelial cell lines, and that its expression was strongly upregulated by several different proinflammatory stimuli. Next, we demonstrated that overexpression of Adrx by transfected HUVEC’s resulted in suppressed TNFα-induced expression of ICAM-1 and VCAM-1 adhesion molecules, reduced monocyte adhesion, and reduced phosphorylation of key signaling molecules in the MAPK and NF-κB pathways. Overexpression of mutated Adrx, which lacked anti-redox activity, was not as effective as native Adrx in suppressing TNFα-induced expression of ICAM-1 and VCAM-1 adhesion molecules. We also demonstrated that knock-down of Adrx expression with siRNA resulted in enhanced expression of ICAM-1 and VCAM-1, and enhanced monocyte adhesion to TNFα-stimulated HUVEC’s. Finally, we demonstrated that Adrx mRNA expression was significantly greater in atheromatous vs. normal tissue from human carotid arteries. These collective results support the conclusion that Adrx functions as a negative regulator of cytokine-induced inflammatory responses in vascular EC, that these effects are mediated by suppression of MAPK and NF-κB signal pathways, and that these anti-inflammatory effects are partially dependent upon the anti-redox activity of Adrx.

Adrx is a 24-kD protein containing a CXXC-type peroxiredoxin-like 2 domain that has redox regulatory activity similar to the members of the peroxiredoxin family. Several different experimental strategies led to the initial recognition of Adrx, and its potential biological significance. Xu *et al*. conducted genome-wide expression screening to identify genes upregulated during M-CSF and RANKL-induced osteoclast differentiation^6^. They cloned a gene whose expression was significantly induced by M-CSF, and named it peroxiredoxin-like 2 activated in M-CSF stimulated monocytes or PAMM (a synonym for Adrx)^6^. More recently, Jedrychowski *et al*. performed proteomic analysis of GLUT4 storage vesicles and identified a protein that they named adiporedoxin (Adrx), because it was preferentially expressed in adipose tissue and contained redox regulatory activity^7^. Similarly, our group identified PAMM or Adrx by searching for genes that were highly induced in mature adipocytes, but not in preadipocytes^8^.

It is evident that Adrx exerts many of its biological effects through inhibition of signal transduction pathways. For example, Xu *et al*. observed that overexpression of Adrx completely abolished RANKL-induced NF-κB and JNK activation, as well as osteoclast formation^6^. Jedrychowski *et al*. observed that Adrx protein levels in human adipose tissues were inversely correlated with the ratio of phosphorylated JNK to total JNK protein^7^. Our group previously demonstrated that Adrx exerted anti-inflammatory effects by suppressing MAPK signaling pathways in macrophages^8^, and in the current study we demonstrated that the anti-inflammatory effects of Adrx on endothelial cells were mediated by suppression of MAPK and NF-κB signal pathways. The mechanisms by which Adrx inhibits MAPK and NF-κB signaling remain unknown. However, it is well established that H_2_O_2_ can function as a second messenger for inflammatory stimuli that trigger NF-κB and MAPK pathways^9^,^10^. It is similarly well established that TNFα-induced ER stress contributes to the activation of both MAPK and NF-κB signaling^11^,^12^,^13^. Jedrychowski *et al*. observed that Adrx is located in the ER membrane, and that it plays an important role in the assembly and secretion of proteins containing disulfide bonds; they hypothesized that Adrx helps control metabolically related ER oxidative stress in adipocytes by facilitating protein folding and secretion under these conditions^7^. Consequently, it is reasonable to postulate that Adrx may inhibit TNFα-induced MAPK and NF-κB activation by attenuating ER stress through its anti-redox activity and ability to promote protein folding and secretion. In support of this concept, Adrx null mice showed increased ER stress, along with decreased adipokines and collagen secretion, resulting in a phenotype that included hyperinsulinemia and decreased adipose fibrosis^7^.

The pathogenesis of vascular inflammatory diseases, such as atherosclerosis, involves deposition of lipids, and interactions between macrophages, endothelial cells, and smooth muscle cells in the vessel wall^14^,^15^. Human obesity, which increases the risk of atherosclerosis, is associated with a low grade of inflammation, caused by the interaction of adipocytes and infiltrated macrophages^16^. We previously demonstrated that high levels of Adrx are secreted by adipocytes, and that this Adrx can suppress the response of macrophages to inflammatory stimuli^8^. In the current study, we have demonstrated that Adrx inhibits endothelial cell activation, an early step in vascular inflammatory disease, and that its expression is significantly increased in human atheromatous tissue^8^. The ability of Adrx to inhibit responses of macrophages and endothelial cells to inflammatory stimuli, in conjunction with its increased expression in human atheromatous tissue suggests that Adrx has considerable potential as a therapeutic anti-inflammatory agent for the treatment of vascular inflammatory diseases.

The expression patterns of Adrx during the course of different stimuli are different, which may be due to the different signaling pathways that govern the expression of Adrx. We observed that the LPS-induced expression of Adrx in HUVECs is different with that in HLMECs. The mechanisms underlying this phenomena need to be further investigated. In addition, it would be interesting to find out if Adrx also help cytokines secretion from macrophages or endothelial cells, and further if Adrx act as an important molecule that coordinates the functional interaction of macrophages and endothelial cells in micro-environment of atherosclerotic lesions.

In summary, our present work has demonstrated that Adrx functions as a negative regulator of cytokine-induced inflammatory responses in vascular EC, that these effects are mediated by suppression of MAPK and NF-κB signal pathways, and that these anti-inflammatory effects are partially dependent upon the anti-redox activity of Adrx.

## Methods

### Cell culture and Reagents

All primary human vascular endothelial cells including HAEC, HCAEC, HDMEC, HLMEC and HUVEC were acquired from Lonza Walkersville Inc. (Walkersville, MD) and cultured in EGM or EGM2 medium according to the manufacturer’s instruction. In all the experiments, cells were used within five passages. The human acute monocytic leukemia cell line THP-1 was obtained from American Type Culture Collection (ATCC) and was grown in RPMI 1640 containing 10% fetal bovine serum. Other cell lines including CHO, Cos-7, NIH3T3, 293 T, HeLa, Jurkat, RAW264.7 and U937 were purchased from ATCC and cultured in DMEM supplemented with 10% fetal bovine serum. Human recombinant TNFα and LPS were purchased from Sigma (Saint Louis, MO). VCAM-1 (sc-13160), ICAM-1 (sc-1511-R), E-selectin (sc-14011), IKKα/β (sc-7607), and actin (sc-1616) antibodies were purchased from Santa Cruz Biotechnology (Santa Cruz, CA). Phospho-JNK, JNK, phospho-IKK, IKK, phosphor-ERK1/2, ERK1/2, phosphor-p38 and p38 antibodies were purchased from Cell Signaling Technology.

### Protein isolation and Western blot

After washing twice with PBS, cells were gently scraped with a rubber policeman into 5 ml of ice-cold PBS, and centrifuged at 1,000 g for 5 min at 4 °C. Cells from each 10-cm dish were then resuspended and lysed in 0.5 ml of lysis buffer containing 50 mM NaH_2_PO_4_, pH 7.6, 250 mM NaCl, 50 mM NaF, 10 mM imidazole, 0.5% Nonidet P-40, 1 μg/ml leupeptin, and 1 mM phenylmethylsulfonyl fluoride. The cell lysate was left on ice for about 20 min and then sonicated and centrifuged at 10,000 g for 10 min at 4 °C. Protein concentrations were determined by the Bradford method (Bio-Rad Laboratories, Hercules, CA), with bovine serum albumin (BSA) as the standard. Proteins (50 μg) were separated by SDS-PAGE and transferred onto nitrocellulose membranes in transfer buffer containing 0.1% SDS. The membranes were blocked with 5% nonfat dry milk in 0.05% Tween 20 in Tris-buffered saline (TTBS) for 2 h and incubated with the primary antiserum at a 1:1,000 dilution in the blocking buffer for 1 h. After being washed with TTBS three times for 10 min each, the membranes were incubated with a 1:2,000 dilution of secondary antibody in TTBS for 1 h. Following three, 10-min washes with TTBS, membranes were incubated with SuperSignal West Pico Chemiluminescent Substrate (Pierce, Rockford, IL) and exposed to X-ray film.

### Quantitative real time RT-PCR (Q-PCR)

After removing the genomic DNA using DNase I (Ambion), 2 μg of total RNA was reverse-transcribed to cDNA using a high-capacity cDNA reverse transcription kit (Life Technologies). QPCR was performed with StepOne Plus real-time PCR system (ABI) using SYBR Green master mix (ABI). Forty cycles were conducted as follows: 95 °C for 30 s, 60 °C for 30 s, preceded by 1 min at 95 °C for polymerase activation. Primer sequences for all genes we measured in this report are available upon request. Quantification was performed by the delta cycle time method, with β-actin used for normalization.

### Transfection

Transient transfection into HUVECs was performed by electroporation following the manufacturer’s instruction (Amaxa, MD). Briefly, HUVECs were grown to confluence in EGM2 complete growth medium. Cells were collected and washed once with medium, and resuspended with the electroporation buffer (Amaxa). After electroporation, cells were plated on 6-well plates and the transfection efficiency was monitored by fluorescent microscopy. Over 60% of the cells were GFP positive.

### Monocyte adhesion assay

HUVECs were transfected with Flag-Adrx or empty vector for 24 h in a 6-well plate and then stimulated with TNFα (10 ng/ml) for 8 h. Medium containing TNFα was removed and fresh medium was added after 2 washes with PBS. On the other hand, THP-1 cells or human primary monocytes (Lonza) were labeled with fluorescein isothiocyanate using a PKH67 fluorescent staining kit (Zynaxis, Inc., Malvern, PA) according to the instructions of the manufacturer for 30 min and gently washed 2 times. Finally, 5 × 10^5^ fluorescence dye-labeled THP-1 cells were added to each well and allowed to interact with HUVECs for 1 h at 37 °C. Unbound THP-1 cells were removed by gently washing with cold PBS. Images were taken using a Cytation 3 Cell Imaging Multi-mode Reader (Biotek Instruments). Adherent THP-1 cells were counted.

### Short-interfering RNA mediated Adrx knockdown

Pre-designed siRNA targeting human Adrx, as well as its negative control, were purchased from Santa Cruz Biotechnology (USA). siRNA was transfected into HUVECs by electroporation following the manufacturer’s instruction (Amaxa, MD). 24 hours later, cells were treated with TNFα (10 ng/ml) for 8 hours. Then cells were harvested and the cell lysate was isolated to assess Adrx knockdown and expression of endothelial adhesive molecules such as ICAM-1 and VCAM-1 by Western blot.

### Human carotid artery sample collection and dissection

Collection of human carotid endarterectomy specimens was approved by the IRB at Greenville Health System, and all patients gave written informed consent. All procedures were performed in accordance with the NIH guidelines and regulations on human subjects. Patients enrolled in the study had greater than 50% stenosis in the carotid artery and a prior stroke or TIA, or greater than 70% carotid artery stenosis. Carotid endarterectomy specimens were obtained from 8 patients at the time of surgery at Greenville Memorial Hospital. The excised specimens were immediately immersed in Belzer UW Cold Storage Solution (Bridge to Life Ltd., Columbia, SC). Intact specimens, ranging from approximately two to five cm in length, were nominally centered around the carotid artery bifurcation and usually contained portions of the common carotid artery, the internal carotid artery, and the external carotid artery. Each specimen was sliced into a series of transverse segments nominally 5-mm thick using a custom slicing device. The slices were dissected by a trained investigator into samples consisting of fibrous cap, atheroma, diseased media, and ‘normal’ tissue, defined as that part of the common carotid artery devoid of any visible atherosclerotic lesion.

### Statistics

Data were expressed as mean ± SD. Statistical analysis between two groups was performed by unpaired Student’s *t*-test. Data from 3 groups and more were analyzed by two-way ANOVA followed by Bonferroni post-test. A value of p < 0.05 was considered statistically significant.

## Additional Information

**How to cite this article**: He, H. *et al*. Adiporedoxin suppresses endothelial activation via inhibiting MAPK and NF-κB signaling. *Sci. Rep.* **6**, 38975; doi: 10.1038/srep38975 (2016).

**Publisher's note:** Springer Nature remains neutral with regard to jurisdictional claims in published maps and institutional affiliations.

## References

[b1] Kinlay S., Libby P. & Ganz P. Endothelial function and coronary artery disease. Curr. Opin. Lipidol. 12, 383–389 (2001). 11507322 10.1097/00041433-200108000-00003

[b2] Glass C. K. & Witztum J. L. Atheroslcerosis: The road ahead. Cell. 104, 503–516 (2001). 11239408 10.1016/s0092-8674(01)00238-0

[b3] Sprague A. H. & Khalil R. A. Inflammatory cytokines in vascular dysfunction and vascular disease. Biochem. Pharmacol. 78, 539–552 (2009). 19413999 10.1016/j.bcp.2009.04.029 PMC2730638

[b4] von der Thüsen J. H., Kuiper J., van Berkel T. J. & Biessen E. A. Interleukins in atherosclerosis: molecular pathways and therapeutic potential. Pharmacol. Rev. 55, 133–166 (2003). 12615956 10.1124/pr.55.1.5

[b5] Rahman A., Kefer J., Bando M., Niles W. D. & Malik A. B. E-selectin expression in human endothelial cells by TNF-alpha-induced oxidant generation and NF-kappaB activation. Am. J. Physiol. 275, L533–544 (1998). 9728048 10.1152/ajplung.1998.275.3.L533

[b6] Xu Y. . PAMM: a redox regulatory protein that modulates osteoclast differentiation. Antioxid. Redox. Signal. 13, 27–37 (2010). 19951071 10.1089/ars.2009.2886 PMC2877117

[b7] Jedrychowski M. P. . Adiporedoxin, an upstream regulator of ER oxidative folding and protein secretion in adipocytes. Mol. Metab. 4, 758–770 (2015). 26629401 10.1016/j.molmet.2015.09.002 PMC4632174

[b8] Guo F. . Adipocyte-derived PAMM suppresses macrophage inflammation by inhibiting MAPK signaling. Biochem. J. 472, 309–318 (2015). 26438880 10.1042/BJ20150019 PMC4754088

[b9] Rhee S. G. . Intracellular messenger function of hydrogen peroxide and its regulation by peroxiredoxins. Curr. Opin. Cell Biol. 17, 183–189 (2005). 15780595 10.1016/j.ceb.2005.02.004

[b10] Wood Z. A., Poole L. B. & Karplus P. A. Peroxiredoxin evolution and the regulation of hydrogen peroxide signaling. Science. 300, 650–653 (2003). 12714747 10.1126/science.1080405

[b11] Galán M. . Mechanism of endoplasmic reticulum stress-induced vascular endothelial dysfunction. Biochim. Biophys. Acta. 1843, 1063–1075 (2014). 24576409 10.1016/j.bbamcr.2014.02.009 PMC4086191

[b12] Lenna S., Han R. & Trojanowska M. Endoplasmic reticulum stress and endothelial dysfunction. IUBMB Life. 66, 530–537 (2014). 25130181 10.1002/iub.1292 PMC4181710

[b13] Lan K. C. . Advanced glycation end-products induce apoptosis in pancreatic islet endothelial cells via NF-κB-activated cyclooxygenase-2/prostaglandin E2 up-regulation. PLoS One. 10, e0124418 (2015). 25898207 10.1371/journal.pone.0124418 PMC4405342

[b14] Libby P. Inflammation in atherosclerosis. Nature. 420, 868–874 (2002). 12490960 10.1038/nature01323

[b15] Hansson G. K. Inflammation, atherosclerosis, and coronary artery disease. N. Engl. J Med. 352, 1685–1695 (2005). 15843671 10.1056/NEJMra043430

[b16] Martinez-Santibañez G. & Lumeng C. N. Macrophages and the regulation of adipose tissue remodeling. Annu. Rev. Nutr. 34, 57–76 (2014). 24850386 10.1146/annurev-nutr-071812-161113

